# 813 Histologic Changes of Skin Biopsies After Autologous Skin Cell Suspension

**DOI:** 10.1093/jbcr/irac012.361

**Published:** 2022-03-23

**Authors:** Shana Lennard, Jeffrey E Carter, Nicole M Kopari, Michael Cook, John Paige, Ian Hodgdon, Herb A Phelan, William L Hickerson

**Affiliations:** University Medical Center New Orleans Burn Center, New Orleans, Louisiana; University Medical Center- New Orleans, New Orleans, Louisiana; Children's Hospital New Orleans, New Orleans, Louisiana; LSUHSC New Orleans, New Orleans, Louisiana; LSUHSC New Orleans, New Orleans, Louisiana; LSUHSC New Orleans, New Orleans, Louisiana; LSUHSC-New Orleans Department of Surgery, New Orleans, Louisiana; Spectral MD, Avita Medical, AccessPro Med, Memphis, Tennessee

## Abstract

**Introduction:**

Over 10,000 cases of autologous skin cell suspension have been performed around the world for the treatment of burn and soft tissue injuries. A key component of the procedure is the harvest of skin biopsies which are exposed to enzymatic degradation. In some regions, epidermal graft harvest has been attempted manually without enzymatic degradation. Our study goal was to examine the histologic changes of the skin biopsies in manual versus enzymatic degradation.

**Methods:**

Our study was an IRB-approved, prospective controlled analysis of residual skin harvested from 10 patients undergoing hernia repair. Two specimens from each patient were procured intraoperatively with each measuring 2x3cm. Each specimen produced two 4mm punch biopsies from three regions (control, mechanical, and enzymatic) for a total of 12 specimens per patient. Enzymatic specimens were prepared using the Avita Medical ReCell® system per manufacture instructions for use. Mechanical specimens were prepared using an abrasive pad until epidermis was macroscopically removed. Histologic analysis was performed with hematoxylin and eosin stain and whole slide scanning. Two or more investigators reviewed each biopsy concurrently with consensus agreement on the remaining epidermis and evidence of degraded reticular dermis. Descriptive statistics were used to assess the variances in the three groups.

**Results:**

The mean residual epidermis was 9% in the enzymatic group, 35% in the mechanical, and 98% in the control. Epidermal harvest was higher in the enzymatic group relative to the mechanical group (two tailed t-test = 0.0008). Reticular dermis was degraded in 10% of the mechanical specimens and none of the enzymatic specimens.

**Conclusions:**

Epidermal harvest was more consistent in the enzymatic group with less trauma to the dermis. Our study suggest that mechanical harvest requires larger donor sites given the decreased epidermal harvest. Further research is needed to determine impact of cell isolation technique on autograft cell suspension viability and distribution of cell types harvested.

